# The impact of the COVID-19 pandemic on the quality and reliability of smoking cessation videos on YouTube: a comparative study across three pandemic periods

**DOI:** 10.3389/fpubh.2025.1675473

**Published:** 2026-01-05

**Authors:** Yağmur Gökseven Arda, Saliha Büşra Aksu, Seda Özmen Sever, Güzin Zeren Öztürk

**Affiliations:** Department of Family Medicine, Istanbul Şişli Hamidiye Etfal Training and Research Hospital, University of Health Sciences, Istanbul, Türkiye

**Keywords:** quit smoking, smoking cessation, YouTube, public health, digital health, JAMA, GQS

## Abstract

**Background:**

YouTube has become a prominent source of health information, particularly during the COVID-19 pandemic. However, the quality and reliability of its content remain variable. This study aims to evaluate the impact of the pandemic on the quality, reliability, and informational structure of smoking cessation videos published on YouTube.

**Methods:**

This comparative descriptive content analysis included 600 YouTube videos collected across three periods: pre-COVID (November 2018–October 2019), mid-COVID (June 2020–May 2021), and post-COVID (June 2023–May 2024). For each period, the top 50 most-viewed and 50 randomly selected videos for the keywords “quit smoking” and “smoking cessation” were analyzed. After applying exclusion criteria, 271 videos were included. Video quality was assessed using the Journal of the American Medical Association (JAMA) Benchmark Criteria and Global Quality Score (GQS), while engagement metrics (view count, like ratio, watch time) were recorded.

**Results:**

Of the 271 videos, 66.8% were classified as useful, while 33.2% were misleading. A significant difference in content quality was observed across the three COVID-19 periods (*p* = 0.017). Videos from the mid-COVID period were significantly more likely to be rated as useful and high-quality compared to the pre-COVID period (*p* = 0.030), reflecting a temporary increase in content reliability during the pandemic peak. Educational content consistently showed the highest quality scores across all periods. In multivariable regression, GQS was the strongest predictor of perceived usefulness (OR for moderate vs. low = 38.9, *p* = 0.001). The logistic regression model demonstrated excellent discriminative performance, with an AUC of 0.912 (95% CI: 0.879–0.946; *p* < 0.001), effectively distinguishing useful from non-useful videos.

**Conclusion:**

The COVID-19 pandemic led to a transient improvement in the quality of smoking cessation content on YouTube, driven largely by contributions from healthcare professionals. However, the post-pandemic decline in content quality underscores the need for sustained digital health strategies and greater professional engagement to promote reliable online health information.

## Introduction

The COVID-19 pandemic has led to profound changes in daily habits, health behaviors, and methods of accessing healthcare services worldwide. During this period, the demand for health-related information increased, and the digital delivery of healthcare services became more widespread (1). Restrictions in access to in-person healthcare and social isolation measures during the pandemic prompted individuals to turn to digital platforms and social media to obtain health information. Among these platforms, YouTube emerged as one of the most popular digital sources due to its ease of access for users seeking health-related information (2).

Smoking cessation is a challenging, multidimensional, and complex process in which access to accurate and supportive information is critically important. Especially during periods of heightened stress, such as the pandemic, digital resources have become increasingly necessary to help maintain motivation and provide support throughout the cessation process (3). On the YouTube platform, smoking cessation videos range widely in content, from personal experience narratives to professional health recommendations. However, there are significant concerns regarding the quality, accuracy, and reliability of digital health content (4, 5).

The COVID-19 pandemic has triggered not only a global health crisis but also a worldwide crisis in the information ecosystem, referred to as an *“infodemic.”* The World Health Organization (WHO) defines an infodemic as an overabundance of information—some accurate, some not—that makes it difficult for individuals to access trustworthy sources and reliable guidance, thereby disrupting informed decision-making processes (6).

Infodemic particularly impedes proper guidance in sensitive areas that require behavioral change, such as smoking cessation, and constitutes a digital threat to public health. Therefore, evaluating the quality and credibility of health-related content on digital platforms is essential to mitigate the effects of the infodemic and enhance the visibility of evidence-based information (7).

The literature suggests that digital health platforms assumed a more central role in the search for health information during the pandemic, but this transition was accompanied by an increase in misinformation and disinformation (8). Several studies have evaluated the impact of the pandemic on the quality of health-related content on YouTube (9, 10). Validated tools such as the Journal of the American Medical Association (JAMA) benchmark criteria and the Global Quality Score (GQS) are widely accepted in the literature for assessing the quality and reliability of YouTube content (11–13).

The aim of this study is to comprehensively examine the effect of the COVID-19 pandemic on the quality, reliability, and content types of YouTube videos related to smoking cessation. A comparative analysis was conducted across three distinct time periods: pre-pandemic, peak-pandemic, and post-pandemic. Through this approach, the study seeks to explore how the pandemic influenced the presentation of digital health information, users’ access preferences, and patterns of information utilization.

## Materials and methods

### Patient and public involvement

No patients or members of the public were involved in this study.

### Search design on YouTube and study setting

This study is a comparative descriptive content analysis aimed at systematically assessing the current status and characteristics of smoking cessation content available on the YouTube platform. To evaluate the impact of the COVID-19 pandemic on the quality of YouTube content related to smoking cessation, three distinct periods were defined:

- Pre-pandemic period: November 1, 2018 – October 31, 2019.
- Peak-pandemic (intermediate) period: June 1, 2020 – May 31, 2021.
- Post-pandemic period: June 1, 2023 – May 31, 2024.

These timeframes were defined according to key milestones announced by the WHO. The intermediate period corresponds to the months following WHO’s declaration of COVID-19 as a global pandemic on March 11, 2020, while the post-pandemic phase begins after WHO declared the end of COVID-19 as a Public Health Emergency of International Concern on May 5, 2023 (14, 15).

**Figure 1:**
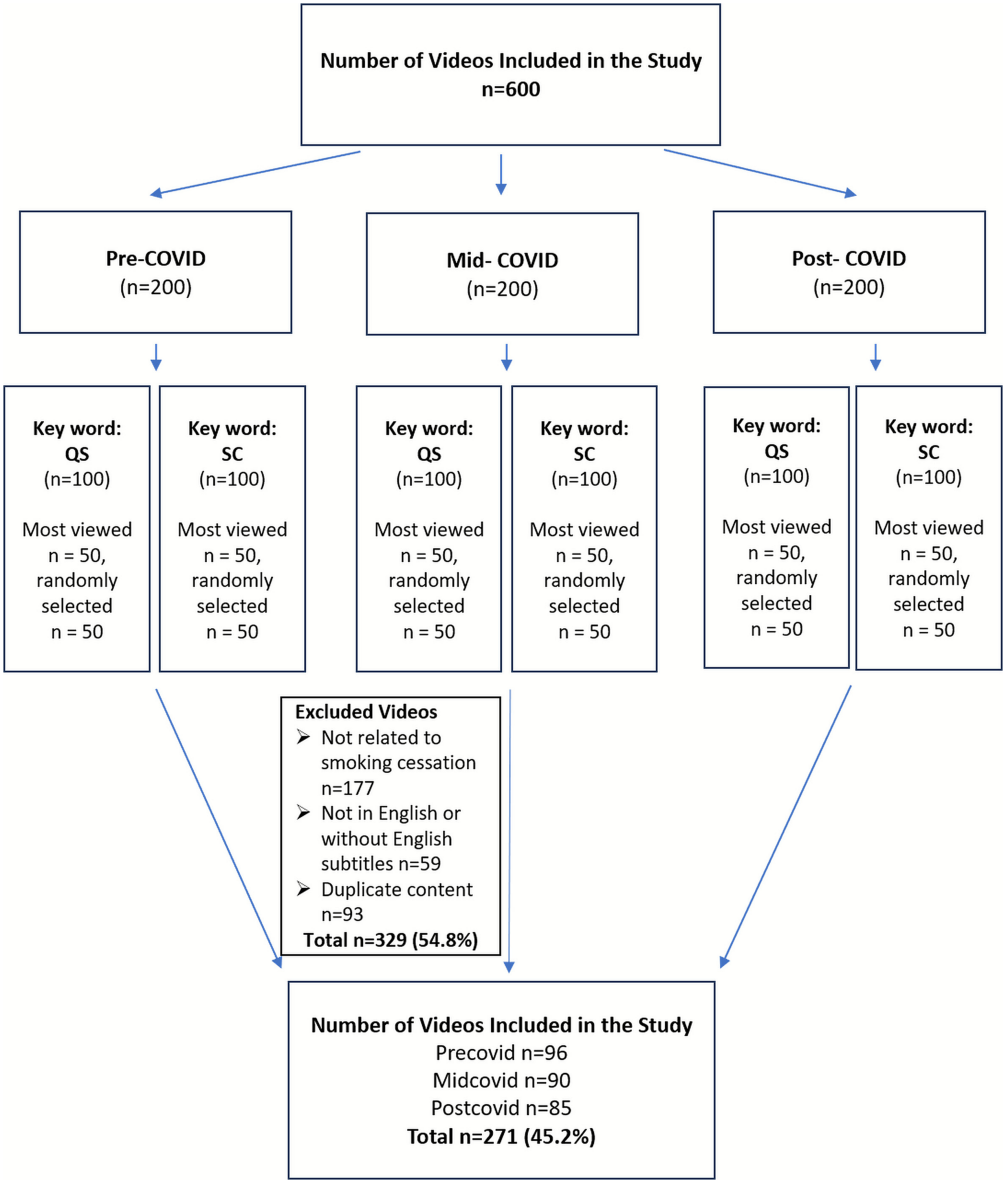
Flow diagram illustrating the search strategy, screening process, and final selection of smoking cessation–related YouTube videos across pre-COVID, mid-COVID, and post-COVID periods. QS, quit smoking; SC, smoking cessation.

For each period, searches were conducted on YouTube using the keywords “quit smoking” and “smoking cessation.” Stratified sampling was applied to ensure proportional representation of videos from each COVID-19 period, minimizing temporal bias and allowing comparability between the “top-viewed” and “randomly selected” groups. For every keyword in each period, the 50 most-viewed videos were selected, along with an additional 50 randomly selected videos, resulting in a total of 100 videos per keyword. Consequently, 200 videos were included for each period, and a total of 600 videos were analyzed across the three timeframes (16, 17) (Figure 1).

All data were retrieved directly through the YouTube Data API v3. Using Python, the top 50 most-viewed videos were selected from the video pool obtained with the ‘viewCount’ sorting parameter, and an additional 50 videos were randomly sampled from the same pool. Due to platform dynamics, API search results vary over time, thus exact reproducibility of the video pool cannot be guaranteed; however, this method helped reduce algorithmic bias and provided a more representative video dataset.

To ensure relevance and consistency, specific inclusion and exclusion criteria were applied. The inclusion criteria were videos in English or with English subtitles, videos directly related to smoking cessation, and with a minimum duration of 1 min. Videos were excluded if they were not in English or lacked English subtitles, were unrelated to smoking cessation, were shorter than 1 min, or represented duplicate content.

For each analyzed video, the following data were recorded: video title, uploader, number of views (NOV), number of comments, watch time of the video, number of channel subscribers, number of likes, number of dislikes, duration the video has been available on the YouTube platform (number of days; NOD), video source, and the type of information provided (17, 18).

All quantitative variables were obtained directly from the publicly available YouTube metadata. Watch time, representing the total duration viewers spend watching a video, used as an indicator of audience retention. Because YouTube’s algorithm prioritizes videos with longer watch times, this variable was included to better assess the relationship between engagement metrics and content quality (19).

Video sources were categorized into four groups:

1. Personal channels.
2. Health professionals (e.g., hospitals, universities, physicians).
3. Health channels (channels providing health information without professional medical credentials).
4. Commercial content (promotional or advertisement-based material).

The type of information provided in each video was classified into three categories based on content:

- Educational content: Videos delivering instructive and informative messages.
- Personal experience: Narratives or testimonials of individuals describing their smoking cessation journey.
- Promotional content: Commercially oriented content.

To assess video popularity and engagement, the following indices were calculated (20):

- View ratio: NOV / NOD.
- Like ratio: (Number of likes × 100) / (Number of likes + Number of dislikes).
- Video Power Index (VPI): (Like ratio × View ratio)/100.

The VPI was calculated to provide a standardized measure of user engagement by combining the like ratio and view ratio of each video. A higher VPI indicates greater audience interaction and perceived popularity. This metric is frequently used in digital media research to capture both qualitative and quantitative aspects of engagement, allowing comparison of content performance beyond raw view counts (20). Although VPI does not directly reflect informational quality, it offers insight into how effectively a video attracts and retains viewer attention, which can influence information dissemination and behavioral outcomes.

### Evaluation of video quality and reliability

All videos were independently evaluated by two physicians certified in smoking cessation. In cases of disagreement, the reviewers discussed the discrepancies to reach consensus. When consensus could not be achieved, a third expert reviewer adjudicated the final categorization (17).

Videos were classified into three quality categories based on the scientific accuracy and comprehensiveness of the content:

- Misleading/Irrelevant information:

Content containing scientifically inaccurate, medically invalid, or unverified claims; off-topic or unrelated material such as general wellness or meditation videos; and videos focused solely on product promotion were included in this category.

- Insufficient but useful information:

Videos that provided potentially helpful tips for smoking cessation but lacked academic or clinical support, or those that included some basic information without covering key elements of the cessation process (e.g., omitting pharmacological treatments or psychosocial support) were placed in this category.

- Excellent information:

Content aligned with recommendations from official health authorities, covering all aspects of smoking cessation (e.g., pharmacotherapy, psychological support, behavioral strategies, motivational elements), with references to healthcare professionals, clinical guidelines, or reputable academic sources. These videos presented evidence-based strategies in a clear and comprehensive manner.

### Assessment of video quality and reliability

To assess the quality and reliability of the videos, both the JAMA benchmark criteria and the GQS were employed (21, 22).

The JAMA benchmark criteria assign one point for the presence of each of the following components, with a total possible score ranging from 0 to 4. A score of 0–1 was considered *low quality*, 2 as *moderate quality*, and 3–4 as *high quality*:

- Authorship: Clear and credible identification of the content author.
- Attribution: All information supported by valid references.
- Currency: Information is up-to-date and consistent with recent medical advances.
- Disclosure: Transparency regarding conflicts of interest or sponsorship.

The GQS is a five-point Likert-type scale based on the overall quality, flow, and usefulness of the video for patient education. A score of 1–2 was considered *low quality*, 3 as *moderate quality*, and 4–5 as *high quality*:

GQS scoring criteria:

1. Poor quality, missing or misleading information, unlikely to be useful for patient education.
2. Sparse content, limited utility, poor presentation.
3. Moderate quality, partially informative, generally adequate technique.
4. Good quality, most important content covered, adequate technique.
5. Excellent quality, comprehensive and highly useful content, adequate technique.

Quality assessments were independently performed by two certified physicians specializing in smoking cessation. Inter-rater agreement was evaluated using Cohen’s Kappa statistic. The inter-rater agreement for overall quality classification was *κ* = 0.87 (*p* < 0.001), indicating excellent agreement. For the JAMA benchmark criteria, *κ* = 0.78 (*p* < 0.001) indicated good agreement. For the GQS assessments, *κ* = 0.86 (*p* < 0.001) demonstrated excellent agreement.

## Statistical analyses

All statistical analyses were performed using SPSS version 25.0. Inter-rater agreement regarding the quality and reliability of the video content was assessed using Cohen’s Kappa statistic. Descriptive statistics were presented as counts and percentages for categorical variables, and as mean, standard deviation, minimum, and maximum values for numerical variables.

The Kolmogorov–Smirnov test was used to assess normality. Chi-square tests were used to compare proportions in independent groups. Since the assumption of homogeneity of variances (one of the fundamental assumptions of classical one-way ANOVA) was violated according to Levene’s test, the Welch ANOVA test, which does not require this assumption, was used to compare the group means. Following the identification of a significant difference between groups, pairwise comparisons were conducted using the Tamhane’s T2 post-hoc test, which is appropriate when variances are unequal. Mann–Whitney U test was used for comparisons between two independent groups when data did not follow a normal distribution. Kruskal-Wallis H test was used for comparisons between three independent groups when data did not follow a normal distribution.

A multivariable logistic regression analysis was conducted to identify factors associated with the usefulness of YouTube videos in the context of smoking cessation. The regression model was built using a manual stepwise approach. Variables that showed statistical significance (*p* < 0.05) in univariate analysis, as well as those considered clinically or theoretically relevant (e.g., JAMA and GQS scores, video watch time, interaction metrics), were included in the model. Prior to model building, multicollinearity among predictor variables was assessed using correlation and variance inflation factor diagnostics, and no multicollinearity concerns were identified.

Model performance and goodness-of-fit were evaluated using standard measures. The Hosmer–Lemeshow test indicated that the model adequately fit the data. Discriminative ability was assessed using ROC curve analysis, which demonstrated that the model had strong classification performance in distinguishing between useful and non-useful videos. Statistical significance was set at *p* < 0.05.

## Results

### Analysis of included videos

A total of 600 videos were screened, of which 271 met the inclusion criteria. 177 videos (29.5%) were excluded for not being related to smoking cessation. Excluded videos were primarily consisted of non-informative or entertainment-oriented content, including smoking initiation pranks made toward family members or friends, marijuana use demonstrations, smoke-blowing scenes, and animated or cartoon materials unrelated to smoking cessation education or public health communication. Additionally, 59 videos (9.8%) were excluded due to the absence of English language or subtitles, and 93 videos (15.5%) were excluded due to duplication (Figure 1).

The largest proportion of videos originated from healthcare professionals (*n* = 92, 33.9%), followed by personal channels (*n* = 83, 30.6%), health-related channels without professional credentials (*n* = 74, 27.3%), and commercial or company-produced videos (*n* = 22, 8.1%). Most videos (98.1%) provided information related to smoking cessation.

Video content quality was categorized as misleading in 90 videos (33.2%) and useful in 181 videos (66.8%). Based on the type of information provided, 115 videos (42.4%) were educational, 56 (20.7%) shared personal experiences, and 100 (36.9%) were promotional or recommendation-based content.

According to JAMA criteria, 142 videos (52.4%) were low quality, 66 (24.4%) moderate quality, and 63 (23.2%) high quality. According to the GQS, 150 videos (25.0%) were low quality, 69 (11.5%) moderate quality, and 52 (8.7%) high quality.

A statistically significant association was found between the type of information and the source of the video (*p* < 0.001). Among educational videos, 64 (55.7%) were provided by healthcare professionals, and 33 (28.7%) by health channels. Of the commercial videos, 36 (36.0%) were uploaded by health channels and 25 (25.0%) by healthcare professionals.

The relationship between video content quality, video type, information type, and interaction metrics—such as NOV, number of comments, NOD, likes, dislikes, subscriber count, and watch time—was evaluated.

According to Mann–Whitney U tests, significant associations were observed between content quality and both video watch time (*U* = 6211.00, *p* = 0.001) and like ratio (*U* = 6350.50, *p* = 0.002). Videos with misleading content had higher mean ranks for both watch time and like ratio compared to useful videos. No significant associations were found between content quality and other variables (*p* > 0.05).

Kruskal–Wallis H analyses showed statistically significant associations between information type and watch time (*p* = 0.023) and like ratio (*p* = 0.005). Personal experience videos had the highest mean rank for watch time, whereas promotional videos had the highest mean rank for like ratio.

A significant association was also found between video source and watch time (*p* < 0.001), with the highest watch times observed in personal experience videos, followed by health channel and healthcare professional videos.

There were statistically significant associations between content quality and both information type (*p* < 0.001) and video source (*p* < 0.001). Among misleading videos, 49 (54.4%) were promotional and 36 (40.0%) were personal experiences. Among useful videos, 110 (60.8%) were educational and 51 (28.2%) were promotional. Regarding source, 45 (50.0%) of misleading videos originated from personal channels and 31 (34.4%) from health channels. Among useful videos, 87 (48.1%) were uploaded by healthcare professionals and 43 (23.8%) by health channels.

Content quality was significantly associated with both JAMA and GQS scores (*p* < 0.001, *p* < 0.001, respectively). Among misleading videos, 80 (88.9%) were rated as low quality and only 3 (3.3%) as high quality according to JAMA. Among useful videos, 60 (33.1%) were high and 62 (34.3%) were low quality. According to GQS, 88 misleading videos (97.8%) were rated as low quality, and only 1 (1.1%) as high quality. Among useful videos, 51 (28.2%) were high and 62 (34.3%) were low quality.

Multivariable logistic regression was used to identify factors associated with the likelihood of a video being classified as useful for smoking cessation. Like ratio was inversely associated with usefulness (OR = 0.98; 95% CI: 0.97–1.00; *p* = 0.045). A moderate JAMA score was significantly associated with higher odds of usefulness compared to low JAMA scores (OR = 3.5; 95% CI: 1.25–9.85; *p* = 0.016). Higher GQS scores were strongly associated with increased likelihood of usefulness: Moderate GQS: OR = 38.9 (95% CI: 4.48–339.33; *p* = 0.001), High GQS: OR = 13.6 (95% CI: 1.02–180.91; *p* = 0.048). Information type significantly influenced usefulness: Personal experience videos were less likely to be rated as useful compared to educational videos (OR = 0.16; 95% CI: 0.04–0.56; *p* = 0.004). Commercial videos were also significantly less likely to be rated as useful (OR = 0.21; 95% CI: 0.06–0.67; *p* = 0.009). Video watch time was not associated with usefulness (*p* = 0.479). The model demonstrated good fit according to the Hosmer–Lemeshow test (*χ^2^*(8) = 10.49, *p* = 0.232), and explained a substantial portion of the variance (Nagelkerke R^2^ = 0.578) (Table 1).

**Table 1:** Multivariable logistic regression analysis of factors associated with the usefulness of YouTube videos on smoking cessation.

Variables	OR (95% Confidence Interval)	*p*-value
Like ratio	0.98 (0.97–1.00)	**0.045**
Watch time (minutes)	0.99 (0.99–1.00)	0.479
JAMA		
Moderate	3.5 (1.25–9.85)	**0.016**
High	1.4 (0.22–9.73)	0.685
GQS		
Moderate	38.9 (4.48–339.33)	**0.001**
High	13.6 (1.02–180.91)	**0.048**
Info type		
Personal	0.16 (0.04–0.56)	**0.004**
Commercial	0.21 (0.06–0.67)	**0.009**

OR, Odds Ratio; JAMA, Journal of the American Medical Association benchmark criteria; GQS, Global Quality Scale.

Reference categories: low JAMA score, low GQS score, and educational videos.

Model fit statistics: Nagelkerke R^2^ = 0.578; −2 Log Likelihood = 198.67; Hosmer–Lemeshow *χ*^2^(8) = 10.49, *p* = 0.232.Values with *p* < 0.05 are presented in bold.

JAMA and GQS scores were moderately correlated (r = 0.76), but VIF analysis (VIF = 1.0) indicated no multicollinearity, supporting their joint inclusion in the model.

The area under the ROC curve was 0.912 (95% CI: 0.879–0.946), indicating excellent discriminative ability of the logistic regression model. The result was statistically significant (*p* < 0.001), suggesting that the model distinguishes well between videos classified as useful and not useful.

### Analysis by COVID-19 periods

**Figure 2:**
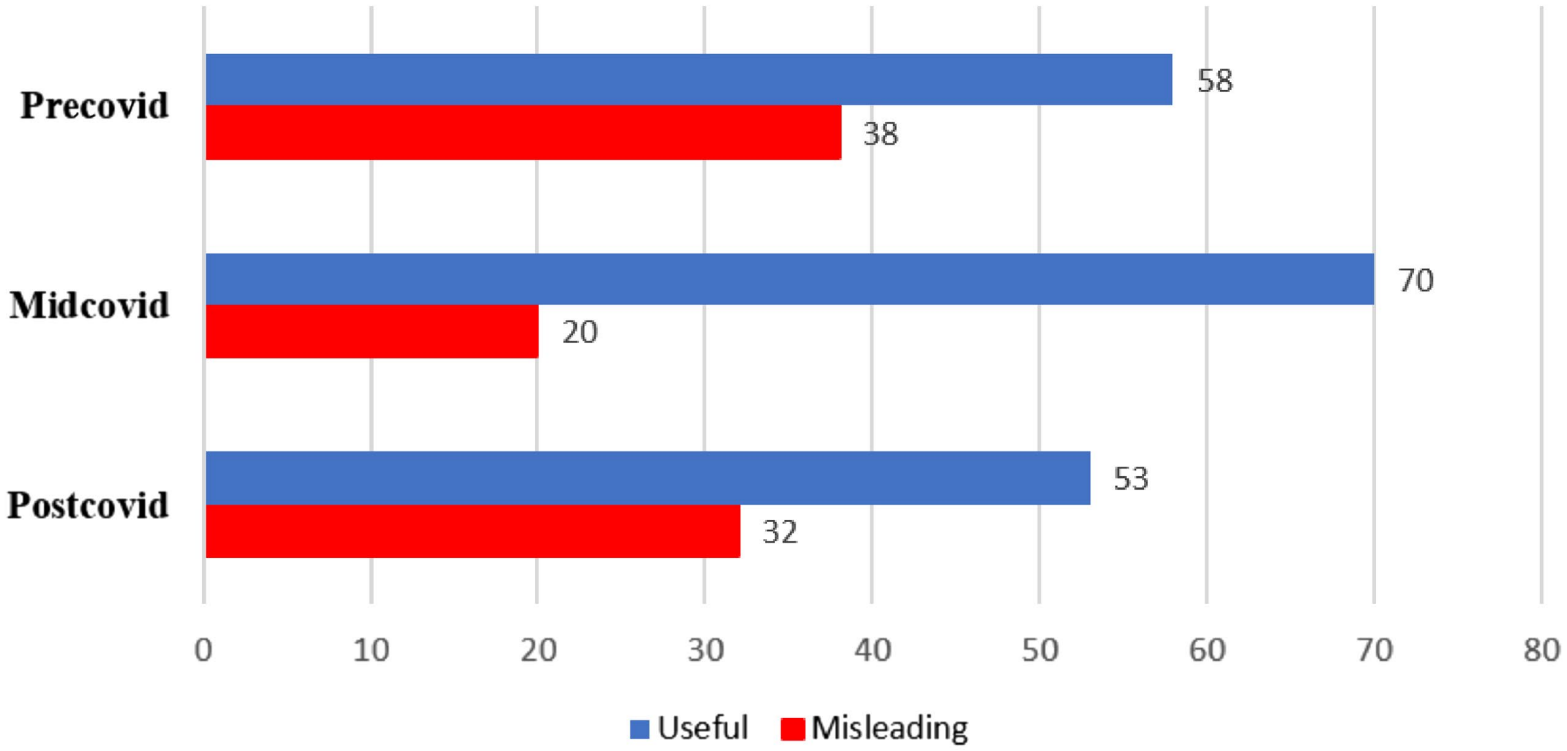
Distribution of misleading and useful video content by COVID-19 periods (*n*) (*p* = 0.017). *Post-hoc* analysis revealed that the content quality of videos published during the mid-COVID period was significantly higher than those published in the pre-COVID period (*p* = 0.030).

When video content quality was analyzed by COVID-19 period, the proportion of videos classified as “useful” was:

- Pre-COVID: 58 videos (32%).
- Mid-COVID: 70 videos (38.7%).
- Post-COVID: 53 videos (29.3%) (Figure 2).

Welch ANOVA revealed a statistically significant difference in content quality between the three periods (*p* = 0.017). Post-hoc analysis indicated that videos published during the Mid-COVID period had significantly higher content quality compared to the Pre-COVID period (*p* = 0.030). However, there were no significant differences between the Mid-COVID and Post-COVID or Pre-COVID and Post-COVID periods (*p* > 0.05).

When comparing video characteristics across the three periods, the pre-COVID period showed a significantly higher mean number of views (NOV) and mean number of dislikes compared to the other periods. (*p* = 0.013 and *p* = 0.001, respectively). Mean like ratio (LR) was significantly higher in the Post-COVID period compared to the others (*p* < 0.001). No significant differences were observed in other video characteristics across periods (*p* > 0.05) (Table 2).

**Table 2:** Viewing and engagement characteristics of videos by COVID-19 pandemic periods.

Variables	Pandemic periods	*n* (%)	95% Confidence interval		Median - IQR	Mean	Std. deviation	*p*-value
			Lower limit	Upper limit				
Number of Views (NOV)	Precovid	96	0	1.758.148,22	4.670,50–120.029,25	797.874,21	4.739.312,69	**0.013**
	Midcovid	90	51.610,66	451.847,68	8.362,50–86.967,75	251.729,17	955.466,13	
	Postcovid	85	0	273.349,75	1.687–12.760,50	131.231,21	658.886,59	
Like	Precovid	96	0	34.435,66	37–2.181,75	15.475,39	93.576,03	0.806
	Midcovid	90	619,00	12.209,99	79–1.257,75	6.414,50	27.670,58	
	Postcovid	85	0	18.047,70	56–428,50	6.962,95	51.390,86	
Number of Comments (NOC)	Precovid	96	0	2.026,56	4–237,75	945,01	5.337,89	0.763
	Midcovid	90	157,36	486,21	4–216,75	321,718	785,04	
	Postcovid	85	40,85	254,67	9–25,50	147,76	495,64	
Watch Time (minutes)	Precovid	96	10,13	62,51	5,35–11,07	22,80	62,51	0.061
	Midcovid	90	11,17	20,46	8,08–13,99	15,82	22,17	
	Postcovid	85	2,65	25,14	5,28–8,56	13,89	52,14	
Dislike	Precovid	96	0	960,23	3–78,75	457,97	2.478,84	**0.001**
	Midcovid	90	39,09	287,34	0–41	163,22	596,63	
	Postcovid	85	0	176,57	0–1,50	80,30	446,31	
View ratio (VR)	Precovid	96	0	879,49	2,47–61,51	401,98	2.356,69	0.703
	Midcovid	90	36,58	338,14	6,12–66,47	187,36	719,91	
	Postcovid	85	20,91	758,85	8,21–46,62	389,88	1.710,62	
Like ratio (LR)	Precovid	96	77,26	90,68	97,34–6,49	83,97	33,12	**<0.001**
	Midcovid	90	63,55	81,49	97,40–100	72,52	42,81	
	Postcovid	85	82,22	95,50	100–1,83	88,86	30,78	
Video Power Index (VPI)	Precovid	96	0	852,67	1,13–57,50	387,36	2.296,48	0.576
	Midcovid	90	33,61	328,26	3,75–58,50	180,94	703,40	
	Postcovid	85	18,36	744,88	7,85–36,21	381,62	1.684,13	

*n*, number of videos.

Group comparisons were performed using the Kruskal–Wallis H test.Values with *p* < 0.05 are presented in bold.

For each COVID-19 period, the relationships between content quality and information type, video source, JAMA score, GQS, watch time, view ratio (VR), like ratio (LR), and video power index (VPI) were evaluated (Table 3). A significant association was found between content quality and information type in all three periods (*p* < 0.001 *p* < 0.001, *p* < 0.001, respectively). Similarly, content quality and video source were significantly associated in all periods (*p* = 0.001 for Pre-COVID, *p* = 0.007 for Mid-COVID, *p* < 0.001 for Post-COVID). In all three periods, JAMA and GQS classifications were significantly associated with content quality (*p* < 0.001, *p* < 0.001, *p* < 0.001, *p* < 0.001, *p* < 0.001, *p* < 0.001, respectively).

**Table 3:** Content quality by information type and video source across the COVID-19 periods.

Variables		Precovid		*p*-value	Midcovid		*p*-value	Postcovid		*p*-value
		Misleading	Useful		Misleading	Useful		Misleading	Useful	
Info type* (*n*, %)	Educational	1 2.6%	28 48.3%	**<0.001**	3 15%	48 68.6%	**<0.001**	1 3.1%	34 64.2%	**<0.001**
	Personal	22 57.9%	7 12.1%		3 15%	9 12.9%		11 34.4%	4 7.5%	
	Commercial	15 39.5%	23 39.7%		14 70%	13 18.6%		20 62.5%	15 28.3%	
Video source* (*n*, %)	Personal channel	24 63.2%	21 36.2%	**0.001**	6 30%	13 18.6%	**0.007**	15 46.9%	4 7.5%	**<0.001**
	Healthcare professional	1 2.6%	22 37.9%		1 5.0%	31 44.3%		3 9.4%	34 64.2%	
	Health channel	9 23.7%	7 12.1%		9 45%	22 31.4%		13 40.6%	14 26.4%	
	Commercial source	4 10.5%	8 13.8%		4 20%	4 5.7%		1 3.1%	1 1.9%	
JAMA	Low	35 92.1%	23 39.7%	**<0.001**	18 90%	20 28.6%	**<0.001**	27 84.4%	19 35.8%	**<0.001**
	Moderate	2 5.3%	20 34.5%		2 10%	21 30%		3 9.4%	18 34%	
	High	1 2.6%	15 25.9%		0	29 41.4%		2 6.3%	16 30.2%	
GQS	Low	37 97.4%	25 43.1	**<0.001**	20 100%	25 35.7%	**<0.001**	31 96.9%	12 22.6%	**<0.001**
	Moderate	1 2.6	24 41.4		0	17 24.3%		0	27 50.9%	
	High	0	9 15.5		0	28 40%		1 3.1%	14 26.4%	
Watch time (mean ± SD)		31.06 ± 58.83	17.39 ± 64.73	**<0.001**	12.60 ± 26.36	16.74 ± 20.95	0.088	27.59 ± 83.60	5.63 ± 6.38	**0.001**
View ratio^ (mean ± SD)		248.56 ± 646.89	502.49 ± 2993.16	0.505	17.65 ± 44.90	235.85 ± 810.68	0.107	577.12 ± 2305.27	276.83 ± 1234.60	0.957
Like ratio^ (mean ± SD)		92.13 ± 22.57	78.63 ± 37.74	0.073	78.56 ± 40.34	70.79 ± 43.62	0.260	96.42 ± 17.64	84.29 ± 35.90	0.114
Video power index^ (mean ± SD)		241.49 ± 628.61	482.93 ± 2917.15	0.799	17.11 ± 43.76	227.74 ± 792.25	0.240	569.22 ± 2281.31	268.35 ± 1201.83	0.723
Total		38	58		20	70		32	53	

*n*, number of videos; JAMA, Journal of the American Medical Association benchmark criteria; GQS, Global Quality Scale; SD, Standard Deviation.

Group comparisons were performed using the Chi-square test (* categorical variables) and the Mann–Whitney U test (^ continuous variables).Values with *p* < 0.05 are presented in bold.

In both Pre-COVID and Post-COVID periods, the mean video watch time was significantly higher for misleading content compared to useful content (*p* < 0.001 and *p* = 0.001, respectively). No significant difference in watch time was observed for the Mid-COVID period (*p* = 0.088). No significant associations were found between content quality and VR, LR, or VPI across any of the three periods (*p* > 0.05).

**Figure 3:**
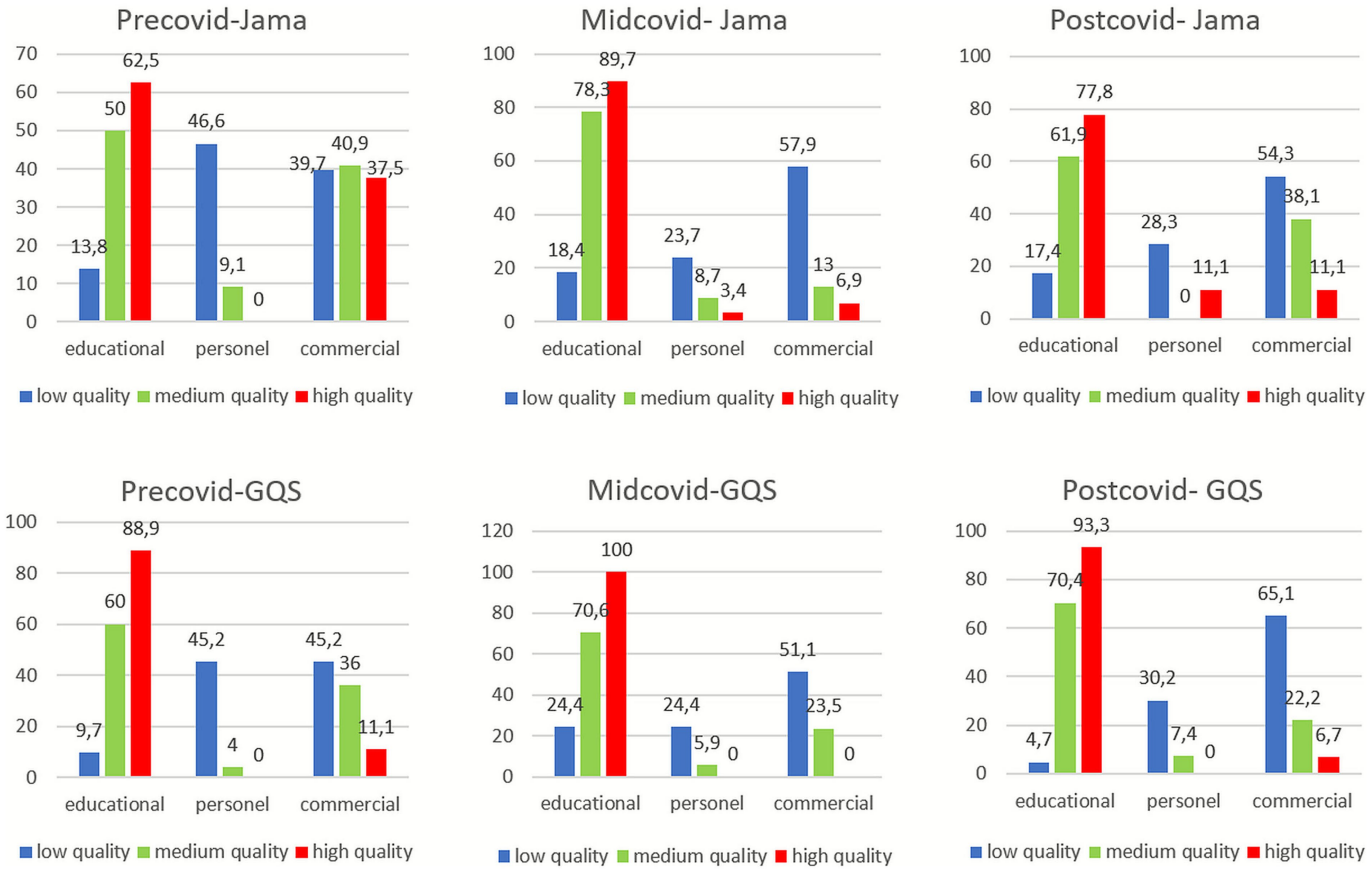
Distribution of JAMA and GQS scores (%) by information type (Educational, Personal, Promotional) for each COVID-19 period. Chi-square.

A statistically significant relationship was found between information type and JAMA quality in all three periods (*p* < 0.001, *p* < 0.001, *p* < 0.001, respectively). In each period, educational content had significantly higher proportions of *high quality* classification compared to personal or promotional content. In the Pre-COVID period, personal content showed the highest rate of *low quality* (46.6%) among all periods. In the Mid-COVID period, educational content showed the highest rate of *high quality* (89.7%) compared to other periods. During the Mid-COVID period, commercial content also had the highest rate of *low quality* (57.9%) (Figure 3).

A significant association was also observed between information type and GQS classification across all three periods (*p* < 0.001, *p* < 0.001, *p* < 0.001, respectively). In all periods, educational content was significantly more likely to be rated as *high quality*, while commercial content had a significantly higher proportion of *low quality*. In the Mid-COVID period, educational content had the highest proportion of *high GQS quality* among the three periods. In the Pre-COVID period, both personal and commercial content showed equal rates of *low quality* (45.2%). In the Post-COVID period, commercial content exhibited the highest proportion of *low quality* (65.1%) (Figure 3).

## Discussion

This study is among the first in the literature to comparatively examine the impact of the COVID-19 pandemic on the quality, reliability, and content type of smoking cessation videos on YouTube across three distinct time periods. Its findings provide valuable insights into how a global public health crisis may influence the nature and quality of digital health information. Previous studies, largely limited to single-period cross-sectional designs, were unable to capture these dynamic changes (4, 5, 18).

The highest proportion of misleading content was observed in the pre-COVID and post-COVID periods, while the mid-COVID period had the highest rate of useful content. In the pre-pandemic period, misleading videos were often characterized as low-quality, personal content uploaded by individual users. In contrast, during the peak of the pandemic, there was a marked improvement in video quality, with a higher prevalence of useful content that was educational in nature and produced by healthcare professionals. This improvement may be partly explained by the public health emergency of the pandemic prompting health authorities to become more involved and visible in combating misinformation through the creation of educational video content. Additionally, platform-level interventions—such as YouTube’s early-pandemic algorithmic adjustments to prioritize content from official health sources like the WHO—may have contributed to increased visibility of high-quality content during this time (23). This suggests that the quality of content on the platform is influenced not only by who produces it but also by shifts in platform-level policies.

Despite the observed improvement in information quality during the pandemic’s peak, the post-COVID period saw a resurgence of misleading videos, many of which were personal, promotional, and low in quality. This regression highlights the impermanence of the improvements and underscores the need for a systematic and sustained approach to digital health information governance. Similar patterns have been reported in other public health topics analyzed on YouTube during the pandemic, including electronic cigarettes and vaccination (23, 24).

Interestingly, during both the pre-COVID and post-COVID periods, misleading videos had longer average watch times than useful videos. In the mid-COVID period, useful videos had slightly longer watch times than misleading ones; however, this difference was not statistically significant. The higher engagement with misleading content in pre- and post-pandemic periods may be attributed to the attention-grabbing, speculative, or anecdotal nature of these videos (25). Such content may be more engaging for viewers and retain their attention longer, despite lacking in quality or accuracy.

The increased viewership of useful content during the mid-COVID period is a positive development; however, the lack of statistical significance suggests that multiple factors, including sample characteristics and viewer behavior, may mediate this effect. These findings imply that beyond the informational quality, elements such as presentation style, structure, and narrative appeal play a role in shaping viewer engagement and content dissemination. For instance, low-quality or misleading videos often employ more emotionally charged titles, simplified explanations, or visually stimulating formats that attract broader audiences, while professionally produced educational content may appear more formal, less entertaining, and thus less engaging to general viewers (20).

This study found that misleading videos scored higher than useful videos in engagement metrics such as view count and like ratio, and that the like ratio was inversely associated with perceived usefulness. This finding suggests that video content quality may not directly correlate with viewer interest and, in some cases, may even exhibit an inverse relationship. A systematic review analyzing diabetes-related videos on YouTube similarly reported that low-quality content attracted higher engagement due to more appealing titles and visuals (26). Other studies in fields such as psychiatry, infectious diseases, otolaryngology, and orthopedic surgery have also demonstrated that low-quality content tends to receive more views and interactions (25, 27–29). Taken together, these findings suggest that engagement-based algorithms may favor popular but low-quality content, potentially increasing the risk of health-related misinformation (30–32).

This engagement-driven imbalance is further compounded by the broad and unfiltered nature of YouTube’s search results. For users seeking smoking cessation–related content, the need to exclude a substantial number of irrelevant videos during the screening process demonstrates the difficulty of accessing accurate health information on open-access platforms. This challenge is particularly concerning when highly engaging misleading content receives algorithmic advantages, potentially exposing users disproportionately to inaccurate, unverifiable, or commercially motivated videos. In a topic like smoking cessation, where behavioral change is critical, the widespread presence of misleading content may hinder individuals from adopting evidence-based health behaviors, and when combined with the longstanding influence of the tobacco industry, may contribute to more substantial public health consequences (33).

Despite the fact that most of the useful and educational videos were produced by healthcare professionals, these videos generally received lower engagement metrics, suggesting that professional sources may struggle with visibility on digital platforms. This finding underlines the need for healthcare professionals and institutions to not only produce accurate information but also develop effective dissemination strategies. This is consistent with findings from other studies. For example, in a study by Loeb et al. on prostate cancer-related YouTube content, videos uploaded by healthcare professionals were found to be of high informational quality but performed poorly in terms of views and engagement (34). Similarly, Batar et al. reported that individuals were more interested in low-quality videos based on patient experiences or commercial promotions than in scientifically sound educational content related to diabetic nutrition (35). These findings underscore the need for healthcare organizations and experts to establish a more visible and strategic presence on digital platforms (27).

Strengthening the regulatory framework governing digital health content is essential to preserve accuracy and ensure user access to trustworthy information. Sustaining the quality improvements observed during the pandemic will require continued collaboration between health authorities, academic institutions, and social media platforms. Establishing permanent verification and labeling systems, refining algorithms to prioritize evidence-based information, and maintaining partnerships with qualified medical content creators may help ensure long-term reliability and reduce the public health risks associated with misleading content (30, 32, 36, 37).

In the multivariable logistic regression model, the GQS emerged as the strongest predictor of a video being perceived as useful for smoking cessation. Videos with moderate and high GQS were found to be 38 and 13 times more likely, respectively, to be rated as useful compared to low-quality videos. This demonstrates the high predictive power of GQS in reflecting content quality and educational value.

Conversely, the JAMA score did not show a strong association with perceived usefulness. Only moderate JAMA scores were statistically significant, while high JAMA scores did not differ significantly from low scores in terms of usefulness. This may reflect that although JAMA criteria assess information credibility, they may not fully capture elements such as behavioral impact or practical usefulness. Nevertheless, multiple studies support the use of validated tools like GQS and JAMA as robust indicators of video quality in the digital health space (9, 13, 16, 38). These findings suggest that such instruments should be widely and systematically integrated into digital health content evaluation.

With regard to information type, educational content was significantly more likely to be considered useful than videos focused on personal experiences or promotional material across all COVID-19 periods. This indicates that information type is a key factor influencing not only content quality but also viewer perception of usefulness in the context of smoking cessation. Educational videos may be more structured, directive, and supportive, making them more effective in guiding behavior change (5).

The inverse relationship between like ratio and perceived usefulness, as well as the lack of association between watch time and content quality, demonstrates the limitations of relying solely on quantitative engagement metrics when evaluating digital health content. The high ROC value obtained for the logistic regression model indicates that the variables used had a strong capacity to distinguish useful videos from non-useful ones. This finding highlights that, beyond quantitative engagement indicators such as views or likes, the qualitative characteristics of video content, including its informational structure and especially quality measures such as the GQS, play an essential role in accurately determining the actual usefulness of videos (37).

This study has several limitations that should be acknowledged. Although efforts were made to achieve broader global representation by including videos with English subtitles in addition to those with English audio, the findings may still not fully reflect the linguistic and cultural diversity of the global audience. Future research should include non-English content to better capture cross-cultural perspectives on smoking cessation communication. In addition, comparative analyses across other visual-digital platforms such as TikTok and Instagram Reels could provide valuable insight into how short-form video formats influence the dissemination and reception of health information.

Although both the top-viewed and randomly selected videos were analyzed to reduce algorithmic bias, the influence of YouTube’s internal ranking and policy filters could not be fully controlled, even when data were retrieved through the YouTube Data API. The randomization process was also limited to the API-generated pool and may not have captured less visible or newly uploaded videos that did not appear within the searchable dataset.

The temporal scope of this study was designed to capture differences across pre-, mid-, and post-pandemic periods; however, seasonal variations or short-term fluctuations in video production and engagement patterns may not have been fully represented.

Finally, engagement metrics such as views, likes, and watch time are indirect measures of user attention and cannot directly assess comprehension or behavioral change. Future studies may incorporate experimental or longitudinal designs to evaluate how exposure to digital health content influences smoking cessation behaviors and long-term information retention.

## Conclusion

This study demonstrates that although there was a temporary improvement in the quality of digital health content during the COVID-19 pandemic, this enhancement was not sustained in the post-pandemic period. While educational videos were found to have higher scores in terms of information quality, misleading content continued to attract greater viewership and engagement, indicating that engagement-based algorithmic systems may inadvertently amplify low-quality or inaccurate information.

Given the substantial influence of health-related content obtained from digital platforms on patient behaviors, content creators and platform providers carry significant responsibility. Health policies should be broadened beyond the realm of physical healthcare services to include the regulation and supervision of digital health environments, promoting the production of public health-oriented content.

The strong predictive performance of the GQS in the logistic regression model demonstrates the value of structured and qualitative assessment tools for distinguishing reliable and useful content. Integrating such validated measures into routine content evaluation processes may enhance the credibility of online health information.

In addition, increasing the visibility and active involvement of healthcare professionals on digital platforms can further support public access to accurate, evidence-based information. Implementing these approaches in combination may strengthen the quality of digital health content and facilitate users’ access to trustworthy resources.

## Data Availability

The original contributions presented in the study are included in the article/supplementary material, further inquiries can be directed to the corresponding author.
